# The Link between HRM Practices and Performance in Healthcare: The Mediating Role of the Organizational Change Process

**DOI:** 10.3390/healthcare11091236

**Published:** 2023-04-26

**Authors:** Cristina Claudia Rotea, Andra-Nicoleta Ploscaru, Claudiu George Bocean, Anca Antoaneta Vărzaru, Mădălina Giorgiana Mangra, Gabriel Ioan Mangra

**Affiliations:** 1 Faculty of Mechanics, University of Craiova, 200585 Craiova, Romania; 2 Doctoral School, University of Craiova, 13 AI Cuza Street, 200585 Craiova, Romania; 3 Department of Management, Marketing and Business Administration, Faculty of Economics and Business Administration, University of Craiova, 13 AI Cuza Street, 200585 Craiova, Romania; 4 Department of Economics, Accounting and International Business, Faculty of Economics and Business Administration, University of Craiova, 13 AI Cuza Street, 200585 Craiova, Romania; anca.varzaru@edu.ucv.ro; 5 Department of Finance, Banking and Economic Analysis, University of Craiova, 200585 Craiova, Romania; madalina.mangra@edu.ucv.ro; 6 Department of Theory and Methodology of Motor Activities, University of Craiova, 200585 Craiova, Romania; gabriel.mangra@edu.ucv.ro

**Keywords:** HRM practices, organizational change, organizational performance, operational performance, employee retention, organizational abandonment

## Abstract

The role of human resources as a change agent in the organizational change process holds great importance. Hence, it is crucial to identify ways human resources can support change. This paper investigates the direct and indirect relationships between human resource management (HRM) practices and organizational performance, as well as the mediating role of the organizational change process in these relationships. The proposed model integrates primary HRM practices, organizational change components, organizational performance, employee retention, and organizational abandonment. We collected data to evaluate the relationships between the model variables through a survey questionnaire applied to 441 Romanian employees in the healthcare industry. The paper used structural equation modeling to test the model’s validity and hypotheses. The results show that HRM practices directly impact organizational performance and have a mediated impact through the organizational change process. Additionally, the direct and mediating effects are consistent, and healthcare employers consider appropriate HRM practices and effective management of the organizational change process as essential drivers to achieve superior performance. The empirical findings provide valuable insights for government policymakers, stakeholders, and health managers on how suitable HRM practices can influence organizational performance.

## 1. Introduction

In today’s rapidly changing economic environment, every organization’s function must demonstrate its positive influence on organizational performance. The function that manages an organization’s most critical resource, human capital, is HRM. Adequate human capital management contributes to achieving organizational objectives [1,2,3,4]. Human resources (HR) are essential in the service sector, which relies heavily on employee skills and competencies. To value these skills and competencies, the organization must implement coherent HRM practices that determine superior organizational performance [5].

Health organizations serve patients who are vulnerable and dependent clients and cannot find health services elsewhere. Therefore, health regulators are working to ensure that health organizations meet patients’ needs more effectively and efficiently [6]. Additionally, despite substantial government investment in public health systems, the increasing demand for health services due to population growth and increased access to health services leads to exponential growth in the health services market.

Any organization operating in the service sector aims to provide quality services at a low cost to demonstrate organizational efficiency. Although delivering their services requires a significant amount of financial resources, the primary resource for health organizations remains human capital due to the unique characteristics of healthcare services, such as the need for employees’ empathy [6]. Inadequate financial and poorly managed human resources can lead to a decline in organizational effectiveness. A performance-oriented approach within healthcare services involves a review of HRM practices to support the organizational change process and achieve performance in terms of financial and human resources. The efficiency-based approach enables the delivery of quality services under cost-minimizing conditions, reducing investments in human resources and leading to staff retention in health organizations, but it can also lead to organizational abandonment [7]. High staff mobility and increasing organizational abandonment can significantly impact user satisfaction and the quality of health services. In light of these issues, healthcare organizations must focus on their workforce adaptability in an uncertain and ever-changing environment. Effective HRM can increase employee retention in health organizations, reduce abandonment rates, and improve patient satisfaction, efficiency, and organizational effectiveness [6,8]. Appropriate HRM practices can also promote staff commitment, leading to an increase in organizational performance [9] and a decrease in organizational abandonment [10].

Recent research in HRM has shown a particular interest in the perceptual approach (based on the Social Exchange Theory), because scholars believe that employee perceptions positively impact attitudes and behaviors that affect organizational performance [4,6,7]. Therefore, implementing appropriate HRM practices leads to better management of human capital, an increase in motivation and commitment within the organization, and an improvement of the organizational culture, ultimately having a positive influence on HR performance indicators (employee retention and organizational abandonment), as well as on operational and financial performance indicators (organizational efficiency and effectiveness). Furthermore, more recent research has addressed the influence of HRM practices on organizational performance during periods of radical change, studying the role of various mediating variables [11]. Therefore, the paper aims to address the literature gap regarding the organizational change process’s mediating role in the relationship between HRM practices and organizational performance, intending to investigate these relationships in the perception of employees in Romanian health organizations. An additional aim was to investigate the relationship between HRM practices and performance in healthcare organizations, focusing on exploring the mediating role of the organizational change process. The study aims to provide insights into the effectiveness of HRM practices in healthcare and identify the factors mediating the relationship between HRM practices and performance. The findings of this study can help healthcare organizations design and implement effective HRM practices and manage the organizational change process to enhance their performance.

The paper’s structure consists of six sections. The first three sections present an introduction, literature review, and methodology, while the last three sections present the research results, discussion, and conclusions.

## 2. Literature Review

In a constantly changing world, organizations must adapt to new market conditions, innovate, and constantly grow to survive and remain competitive. To meet these challenges, many organizational leaders choose to implement change. However, implementing change can be a difficult and complex process that affects organizational performance, employee retention, and abandonment. Therefore, evaluating how HRM practices influence the organizational change process, organizational performance, employee retention, and organizational abandonment is essential. HRM practices are activities that manage employees’ competencies to increase work productivity [12]. As a result, HRM practices can increase organizational effectiveness through recruitment and selection, evaluation, training, reward, employee involvement, and appropriate labor relations [13]. Organizational performance is a complex, multidimensional construct that defines how an organization meets and exceeds its targeted objectives [4]. Over time, various researchers have defined organizational performance differently, using different approaches and perspectives specific to other fields of economic sciences. For example, Zhang et al. [14] proposed defining organizational performance as the measure of achieving organizational objectives. Dryer and Reeves [15] proposed a multidimensional approach to performance, proposing measures for four components of performance: HR performance (employee satisfaction, employee retention, and organizational abandonment), operational performance (efficiency, effectiveness, physical productivity, product quality, and customer satisfaction), financial performance (profit, turnover, return rates), and market performance (share price, return on capital). Later, other researchers showed that not only the financial measures offered by financial accounting should be used. Therefore, organizations must use the non-financial measures offered by managerial accounting, restructured to consider non-financial aspects of organizational activity, particularly in the context of digital transformation, which allows the processing of a large volume of data instantaneously with the help of artificial intelligence [16,17].

Healthcare organizations’ characteristics can influence how HRM practices impact organizational performance [18,19]. According to Martins et al. [6], managing healthcare organizations is complex due to their multiple missions and most employees being medical professionals. Therefore, effective HRM practices can encourage change-oriented behavior, increase employee motivation and morale, and ultimately influence efficiency, effectiveness, and organizational performance [20].

The two indicators that illustrate non-financial performance related to human resources, employee retention, and organizational abandonment are measures of HR performance that have opposing contributions to the organization’s overall performance [21,22]. Organizational abandonment refers to the staff’s willingness to leave the organization for a new job with greater satisfaction and benefits [23]. Organizational abandonment has antecedent variables, primarily HRM practices, which can be added to organizational support, culture, and commitment [24].

Healthcare organizations must implement effective HRM policies to ensure employee retention and prevent organizational abandonment. The loss of employees does not only refer to the individuals, but also to their skills, abilities, and knowledge [25]. High employee retention rates and low organizational abandonment increase organizational performance [26]. Sound HRM practices increase organizational commitment and involvement, including in the organizational change process, reducing abandonment [27,28]. Conversely, dissatisfaction with HRM practices can lead to increased organizational abandonment and reduced efficiency and effectiveness [8]. According to the Resource-Based View (RBV) theory, a firm’s resources and capabilities can contribute to its competitive advantage and overall performance [4,5,13]. In the healthcare sector, HRM practices, such as employee recruitment, selection, training, and development, can be regarded as valuable resources. Such practices can assist healthcare organizations in cultivating a skilled and motivated workforce, resulting in enhanced performance [15]. Additionally, the Social Exchange Theory proposes that employees’ attitudes and behaviors depend on how they perceive their employment relationship with the organization [13]. Therefore, HRM practices significantly affect employees’ perceptions, influencing their involvement in the change process and promoting more significant efforts toward achieving higher performance.

Based on these theoretical considerations, the paper proposes the following hypothesis investigated in the empirical study:

**Hypothesis**  **H1**: *Employees’ perception of healthcare organizations shows that HRM practices positively influence operational performance and employee retention and negatively influence organizational abandonment.*

In organizations, unexpected events may disrupt usual activity, requiring an immediate reaction that affects the normal production process of goods or services. For instance, in the last four years, events in public health policy (the COVID-19 pandemic) and international politics (the war in Ukraine) have affected supply chains, production processes, and tools used in production processes, accelerating the pace of digital transformation in the economy. In addition, inflation, threats in the labor market generated by digital technologies, and difficulty accessing financing are only a few of the changes organizations have faced in recent years.

An increasing number of researchers have investigated the impact of the COVID-19 pandemic on social and economic relations to assess how organizations have reacted to unplanned organizational changes. Worley and Jules [29] showed that most organizations were not prepared for a crisis when the COVID-19 pandemic occurred. That is why researchers such as Amis and Janz [30] have highlighted the need to create an adaptive environment at the organizational level that allows for the continuation of operations under intense stress and the achievement of organizational objectives. Although there is extensive literature on organizational change planning, Kotter [31] highlights that organizations fail to manage planned changes because they do not pay enough attention to the change preparation process, especially concerning HRM practices [32]. While employees implement the changes, they must be involved in the change planning, implementation, and evaluation processes [33]. Since there are multiple variations in how change manifests and affects organizational processes, a unitary model of organizational change cannot be established [34]. Whatever model is chosen must be adequately communicated to obtain the involvement of the organization’s HR in the change process implementation [35,36,37]. Employees must be convinced that change is possible and will bring personal and organizational benefits. Change usually involves new strategies and procedures that can only be applied when the change is accepted and there is a change in attitude that influences employees’ behaviors. Providing explanations of the change’s causes and its positive effects can convince employees to accept the change plan and strive to increase organizational performance [38,39,40].

Based on these theoretical considerations, the paper proposes two hypotheses that the empirical study investigates:

**Hypothesis**  **H2**: *Employees’ perception of healthcare organizations shows that HRM practices positively influence the organizational change process.*

**Hypothesis**  **H3**: *Employees’ perception of healthcare organizations shows that the organizational change process positively influences operational performance and employee retention and negatively influences organizational abandonment.*

Research extensively covers the relationship between HRM practices and organizational performance [41]. For instance, Tortia [42] studied the relationship between HRM practices and organizational performance, using employee wellbeing as a mediating factor. The success of the change process depends on the management’s preparedness for the changes, allowing for the adaptation of interventions and proactive corrections in the change process. However, in today’s economic and public health environment, simplistic, linear planning models are no longer sufficient for planning and implementing changes [40]. Furthermore, untimely change needs may arise that do not fit into organizational change plans. As a result of the changing environment, multiple change and organizational development initiatives may occur, which can overlap, putting additional pressure on organizations.

Change is a constant in organizations today, with operations and strategies always subject to change. As a result, numerous researchers have investigated the change process, its antecedents, its impact on organizational performance, and the role of the mediator of organizational change [43,44]. This paper aims to study the mediator role of the organizational change process in the relationship between HRM practices, organizational performance, employee retention, and organizational abandonment.

The literature review indicates that organizational change can significantly impact organizational performance by improving the efficiency and effectiveness of operations and encouraging innovation and adaptability to environmental changes. Moreover, appropriate HRM practices play an essential role in organizational performance, and can thus improve organizational performance [13,45].

Organizational change can significantly impact organizational performance, but its effect may vary depending on the nature and purpose of the change and how organizations manage it [4,40]. Additionally, organizations should consider communicating openly and transparently with employees and supporting them to develop their skills and competencies to adapt to organizational changes and contribute to their success [6]. Besides the impact on employee retention and organizational abandonment, organizational change can significantly affect other critical organizational performance variables, such as efficiency, effectiveness, and profitability [46].

Based on these theoretical considerations, the paper proposes the fourth hypothesis investigated in the empirical study:

**Hypothesis**  **H4**. *Organizational change significantly mediates the relationship between HRM practices, operational performance, employee retention, and organizational abandonment in the perception of healthcare organizations’ employees.*

## 3. Methodology

Researchers widely discuss the relationships between HRM practices and organizational performance in managerial literature. These practices relate to individual and organizational performance [6,40,41,47,48,49,50,51,52,53,54]. Through a literature review investigating these relationships [6,40,41,48,49,50,51,52,53,54], we selected the following HRM practices for our empirical study: recruitment and selection, training and development, evaluation, reward, and employee involvement. To measure organizational performance, we chose a multidimensional construct that includes operational performance (defined by efficiency and effectiveness) and human resources performance (defined by employee retention and organizational abandonment) [6,40,41,47,48,49,50,51,52,53,54].

**Figure 1:**
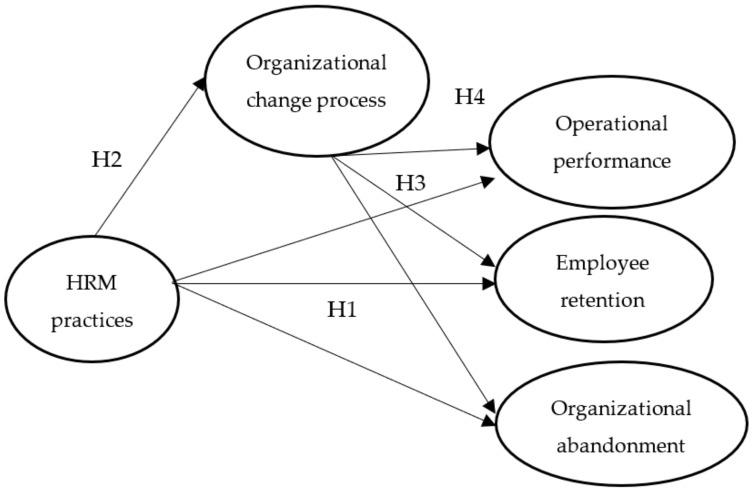
Conceptual model. Source: developed based on literature review.

Using these two opposite concepts that characterize HR performance reduces the risks of common method bias in the questionnaire [55]. Figure 1 illustrates the proposed model, the relationships between the variables, and the research hypotheses.

We utilized structural equation modeling (SEM) to investigate the direct relationships and the mediator role of the organizational change process in the connections between HRM practices and organizational performance. SEM enables the evaluation of relationships between latent variables (unobservable or endogenous to the model), constructed based on observable exogenous variables. Table 1 presents the latent variables, the questionnaire items representing exogenous variables, and the measurement scales. We utilized five-level Likert scales to measure the variables describing HRM practices, an organizational change process, and organizational performance.

To empirically investigate the hypotheses of the proposed theoretical model, we conducted a questionnaire-based survey of employees. The sample comprised 441 employees from Romanian health organizations. We assured respondents of the confidentiality and anonymization of their identity and their organizations. Additionally, we provided an informed consent form along with the questionnaire. The questionnaire included items on personal perceptions without soliciting personal or confidential information about the employing organization. The sampling uses a layered random method, considering three socio-demographic variables: gender, age, and education. Table 2 presents the frequencies of socio-demographic variables.

Table 3 displays the model’s descriptive statistics of the exogenous variables (questionnaire items).

Self-administered questionnaires can lead to a common method bias [55]. To address this issue, we employed Harman’s one-factor principal component analysis test to test the variable set. The factor analysis results revealed that the total variance extracted was below 50% (48.013%), and no significant bias effects were present [55].

## 4. Results

**Figure 2:**
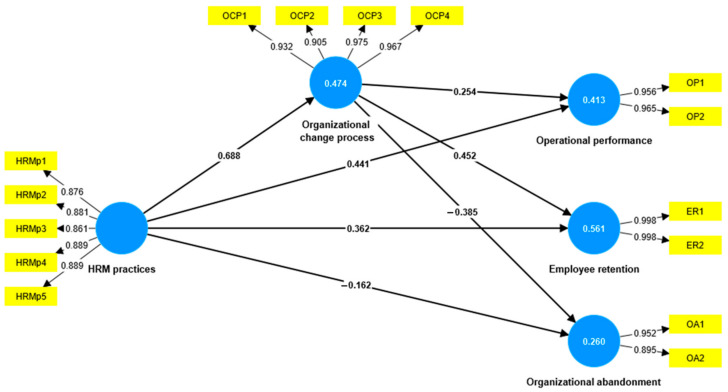
Empirical model. Source: developed by authors using SmartPLS v.3.

According to Hair et al. [56], we employed a PLS algorithm for the SEM model constructed based on the theoretical model. As a result, we obtained a diagram that depicts the relationships between exogenous and endogenous variables, and among endogenous variables. Moreover, we used a reflexive model, whereby the observable variables of the model describe the characteristics of the latent variables. Figure 2 presents the empirical model.

**Figure 3:**
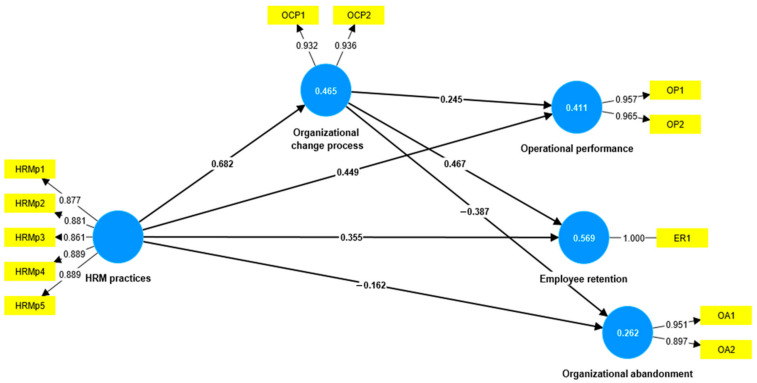
Empirical model modified. Source: developed by authors using SmartPLS v.3.

Considering the multicollinearity of some variables (VIF > 5) in the initial model, we dropped three exogenous variables: ER1, OCP3, and OCP4. Figure 3 illustrates the model obtained after removing these three variables.

Table 4 shows the variance inflation factor (VIF) for the exogenous variables of the modified model. The VIF coefficients have values below 4.0, which eliminates the multicollinearity issue.

The reliability of the model is excellent, with reliability measures above 0.8 (Cronbach’s alpha), 0.8 (composite reliability), and 0.7 (average variance extracted). In addition, we tested the model fit, and the result was good (SRMR = 0.056 < 0.08) (Table 5).

The model displays good discriminant validity according to the Fornell–Larcker criterion, with the values of the main diagonal exceeding all values in the corresponding line and column [57] (Table 6). Additionally, the heterotrait-monotrait (HTMT) ratios recorded were values below 0.85, showing excellent validity (Table 6).

We obtained the path coefficients of the model after applying a bootstrapping procedure (Table 7). According to Hair et al. [56], the relationships described by the model are significant (*p* < 0.005, *t* > 1.6).

Table 7 presents the direct relationships within the empirical model. This table illustrates significant positive direct relationships between HRM practices, operational performance, employee retention, and organizational abandonment. Among these relationships, the HRM practices significantly influence the organizational change process (c = 0.682, *p* < 0.001). On the other hand, HRM practices negatively impact organizational abandonment. The first three research hypotheses are validated. According to the perception of healthcare organizations’ employees, HRM practices positively affect operational performance, employee retention, and organizational change process and negatively impact organizational abandonment. Similarly, organizational performance positively influences operational performance and employee retention and negatively impacts organizational abandonment.

Table 8 presents the specific indirect and total effects of HRM practices on organizational performance after applying the bootstrapping procedure.

The bootstrapping procedure generated significant (*p* < 0.001) indirect effects mediating the relationship between HRM practices and organizational performance, with moderate coefficient values. A comparison between indirect and total effects indicates a significant mediating role played by the organizational change process in the links between HRM practices, operational performance, employee retention, and organizational abandonment. Therefore, the analysis of the total and indirect effects in Table 8 supports validating Hypothesis H4.

## 5. Discussion

The managerial literature that investigates the relationship between HRM practices and organizational performance is extensive [6,45,46,52,58,59,60,61], and the healthcare field presents several particularities that require different approaches [62,63,64]. The strategic dimension of the HR function within healthcare organizations makes HRM practices a crucial antecedent of organizational performance. Health organizations focus on increasing organizational performance through appropriate HRM practices amid continuous changes in this field [46].

We tested the validity of four hypotheses, considering the mediator role of the organizational change process in investigating direct and indirect relationships between HRM practices and organizational performance.

Analyzing the validity of hypothesis H1, we found, in line with other researchers [6,12,41,46,53,54], that there is a direct and significant relationship between HRM practices and organizational performance, employee retention, and organizational abandonment. Specifically, HRM practices, such as recruitment and selection, training and development, performance appraisal, and reward, significantly and positively influence organizational performance and employee retention, and mitigate organizational abandonment. Additionally, research findings indicate that organizational change is essential in mediating the relationship between HRM practices and organizational performance, employee retention, and organizational abandonment. Therefore, managers should pay special attention to organizational changes in healthcare organizations and implement HRM practices that support these changes. Our research suggests that HRM practices and organizational change are critical in increasing performance and employee retention while reducing organizational abandonment. Consequently, organizations should be aware of these relationships, implement HRM practices, and manage organizational change to support organizational performance and success.

Analyzing the validity of hypothesis H2, we found that HRM practices significantly influence organizational change, which confirms the findings of other research [4,32,40]. These results suggest that HRM practices are essential for organizational change, but a robust organizational culture and loyal employees are needed to communicate the effects of these practices. Therefore, healthcare organizations should consider these aspects when developing their HRM practices to achieve successful organizational change. In conclusion, this research supports the hypothesis that there is a significant relationship between HRM practices and organizational change processes. Therefore, organizations should pay more attention to HRM practices and develop them in a way that supports achieving successful and sustainable organizational change.

After investigating hypothesis H3, we discovered a direct relationship between the organizational change process and organizational performance, similarly to the conclusions of other authors [4,31,32,40,43]. Successful changes have been linked to improved organizational performance, while unsuccessful changes have led to decreased performance. This relationship can be explained by the fact that the change process can enhance organizational processes and practices, thus increasing productivity and efficiency. For instance, a change that improves communication and collaboration between departments can reduce errors and processing time, thereby enhancing organizational performance.

Moreover, organizational change can significantly impact employee retention. Successfully managing changes and involving employees in the process can positively affect retention. However, changes imposed on employees without proper explanation or involvement can increase organizational abandonment and decrease retention. For example, a change involving a reduction in working hours may be perceived negatively by employees who may feel that their efforts are not valued and may be tempted to leave the organization. To avoid organizational abandonment, organizations should improve employee engagement and satisfaction by enhancing the work environment and creating a positive and motivating atmosphere through the change process [4,31,32,40,43]. Effective and coordinated implementation of organizational change can also increase engagement and satisfaction, making employees less likely to leave the healthcare organization. Organizations can achieve long-term success and stability in the competitive market by improving these aspects.

Therefore, organizations should invest in organizational change development and change management programs, considering strategies to improve employee retention and reduce organizational abandonment, such as enhancing career development programs, improving the work environment, and increasing employee satisfaction. By implementing these strategies, organizations can benefit from better performance, higher employee retention, and reduced costs associated with organizational abandonment [65,66,67].

After researching hypothesis H4, we concluded that HRM practices and organizational change are two critical antecedents of organizational performance, and their relationship can be complex and multidirectional. Similar to findings from other research [4,6,31,32,40,43,52], we found that HRM practices significantly positively affect organizational performance in healthcare organizations, mediated by organizational change. Specifically, effective HRM practices can contribute to the success of the change process, thus improving organizational performance. At the same time, organizational change can provide an opportunity to enhance HRM practices by improving communication and employee involvement in the decision-making process. Therefore, organizations must consider these interactions and take steps to minimize the adverse effects of change on employees.

Change management components contribute significantly to higher organizational performance, an improved quality of health services, and increased satisfaction among health service users. Additionally, our empirical investigation found that the direct effect between HRM practices and organizational performance has a greater amplitude than the effect mediated through the organizational change process. However, the mediation effect is also important and significant, highlighting the need to emphasize the role of HR in change management to improve organizational performance, consistent with other research findings [65,66,67].

### 5.1. Theoretical Implications

Multiple studies in managerial literature have analyzed the relationships between HRM practices and organizational performance. Still, their results are not always precise due to the lack of consideration of factors related to organizational change [41]. Health organizations comprise personnel with various specialties, not just medical, who interact and interconnect within work teams to provide health services [68]. Due to the dynamic environment and public health problems, such as epidemics or pandemics, health organizations experience systematic organizational change processes [69]. Therefore, healthcare organizations must effectively and efficiently manage organizational change by utilizing appropriate HRM practices to improve patient health. Like Bolton et al. [70], we have demonstrated that HRM practices in healthcare organizations influence performance and several indicators related to HR performance. Sound HRM practices lead to increased performance in all aspects and reduced organizational abandonment [27,28].

The paper reveals significant relationships between HRM practices, operational performance, and HR performance measures. Despite being a significant concern for HR professionals, little research has explored the link between HRM practices and performance in the healthcare sector. The paper’s conclusions indicate that HRM practices are associated with the indicators of organizational performance, such as efficiency, effectiveness, employee retention, and organizational abandonment, which ultimately affect patient care. Furthermore, utilizing the perceptual approach, which allows assessing health employees’ perceptions of HRM practices and organizational performance, can provide insights into how these concepts relate [46] directly and through mediating change processes.

### 5.2. Managerial Implications

Although empirical research has focused on the relationships between HRM practices and organizational performance in recent years, the theoretical underpinnings of existing research have been criticized by researchers, who call for new models to support the evidence of these relationships [6]. Nevertheless, Wright and Haggerty [71] have called for research to investigate the personal and organizational resources that make the organization perform better, especially in a changing and dynamic environment.

The main conclusion of this paper is that HRM practices have a significant impact on organizational performance, with the direct effect between the two variables being more significant than the mediated effect. These research findings have important implications for managerial practice within healthcare organizations. The strategic approach of HRM practices through the organizational change process has shown that the influence of HRM practices on organizational performance can be improved, even in healthcare organizations [72,73,74]. The increasing pace of change affects attitudes and behavior patterns, but through appropriate HRM practices, the organization can carry out efficient and practical activities, ensuring increased employee retention and reduced organizational abandonment.

### 5.3. Limitations and Further Research

Although the theoretical and empirical literature provides valuable insights, this paper has several limitations. Firstly, the empirical study adopts a cross-sectional approach that prevents effects analysis over time. However, given the uncertainties and rapid changes in the current economic environment, a longitudinal study is desirable to evaluate the trends in these relationships. Secondly, employees filling out a self-administered questionnaire about their activities may introduce bias to the research process. Therefore, the paper employs methodological and statistical recommendations to mitigate common method biases [55]. Thirdly, the study can be expanded by incorporating other variables related to the three constructs (HRM practices, organizational change process, and organizational performance), such as organizational culture, employee satisfaction, and commitment. Further research could potentially use these variables as mediators or moderators.

## 6. Conclusions

Our research findings suggest that implementing effective HRM practices and managing organizational change are crucial in enhancing organizational performance while reducing employee turnover and organizational abandonment. Thus, our paper emphasizes the importance of organizations being aware of these connections and taking necessary actions to improve performance. In the healthcare sector, our findings indicate that the organizational change process can mediate the relationship between HRM practices and organizational performance. Employee involvement, combined with well-planned change management training, can enhance the performance of healthcare organizations.

## Figures and Tables

**Table 1:** Questionnaire structure and item scales.

Latent Variable	Item	Scales
Demographic variables	Gender	Male (1), Female (2)
	Age	18–30 years (1), 31–40 years (2), 41–50 years (3), 51–60 years, over 60 years (4)
	Education	High school (1), Bachelor (2), Master (3), Ph.D. (4)
Human resource practices	Recruitment and selection (HRMp1)	Very good (5) Good (4) Moderate (3) Weak (2) Very weak (1)
	Training and development (HRMp2)	
	Evaluation (HRMp3)	
	Rewarding (HRMp4)	
	Employee involvement (HRMp5)	
Organizational change process	Change planning (OCP1)	
	Communicating change (OCP2)	
	Implementing change (OCP3)	
	Assessing change (OCP4)	
Operational performance	Efficiency (OP1)	Very high (5) High (4) Moderate (3) Small (2) Very small (1)
	Effectiveness (OP2)	
Employee retention	Satisfaction with current work, including reward (ER1)	
	Satisfaction with career development in the organization (ER2)	
Organizational abandonment	Intention to leave the organization (OA1)	
	Not recommending the organization to others (OA2)	

Source: developed by authors using SPSS v27.

**Table 2:** Frequencies of socio-demographic variables.

Variable	Answer Options	Frequency	Percent
Gender	Male	174	39.5
	Female	267	60.5
Age	18–30 years	24	5.4
	31–40 years	150	34.0
	41–50 years	159	36.1
	51–60 years	69	15.6
	Over 60 years	39	8.8
Education	High school	36	8.2
	Bachelor	213	48.3
	Master	180	40.8
	PhD	12	2.7

Source: developed by authors using SPSS v27.

**Table 3:** Descriptive statistics.

Variable	N	Min	Max	Mean	Std. Deviation	Skewness	Kurtosis
Recruitment and selection (HRMp1)	441	2	5	4.29	0.595	−0.389	0.458
Training and development (HRMp2)	441	2	5	4.20	0.670	−0.400	−0.224
Evaluation (HRMp3)	441	3	5	4.44	0.608	−0.575	−0.588
Rewarding (HRMp4)	441	3	5	4.16	0.568	0.007	−0.120
Employee involvement (HRMp5)	441	2	5	4.04	0.680	−0.312	0.013
Change planning (OCP1)	441	2	5	4.28	0.781	−0.705	−0.479
Communicating change (OCP2)	441	2	5	3.81	0.993	−0.030	−1.351
Implementing change (OCP3)	441	2	5	4.19	0.760	−0.428	−0.858
Assessing change (OCP4)	441	3	5	4.22	0.752	−0.382	−1.150
Efficiency (OP1)	441	3	5	4.39	0.577	−0.301	−0.740
Effectiveness (OP2)	441	2	5	4.31	0.635	−0.523	0.134
Satisfaction with current work, including reward (ER1)	441	2	5	4.27	0.599	−0.371	0.433
Satisfaction with career development in the organization (ER2)	441	2	5	4.27	0.590	−0.356	0.514
Intention to leave the organization (OA1)	441	1	3	1.16	0.422	2.611	6.390
Not recommending the organization to others (OA2)	441	1	3	1.07	0.301	4.814	24.126

Source: developed by authors using SPSS v27.

**Table 4:** Variance inflation factor.

Variable	VIF
ER1	1.000
HRMp1	3.673
HRMp2	3.638
HRMp3	2.648
HRMp4	3.749
HRMp5	3.645
OA1	2.059
OA2	2.059
OCP1	2.248
OCP2	2.248
OP1	3.527
OP2	3.527

Source: developed by authors using SPSS v27.

**Table 5:** Model reliability.

	Cronbach’s Alpha	Composite Reliability	Average Variance Extracted (AVE)
HRM practices	0.927	0.93	0.773
Operational performance	0.917	0.924	0.923
Organizational abandonment	0.835	0.923	0.854
Organizational change process	0.96	0.961	0.893

Source: developed by authors using SPSS v27.

**Table 6:** Discriminant validity.

Fornell–Larcker Criterion	Employee Retention	HRM Practices	Operational Performance	Organizational Abandonment	Organizational Change Process
Employee retention	1.000				
HRM practices	0.673	0.88			
Operational performance	0.742	0.615	0.961		
Organizational abandonment	−0.56	−0.426	−0.433	0.925	
Organizational change process	0.709	0.682	0.55	−0.498	0.934
Heterotrait-monotrait (HTMT) ratios	Employee retention	HRM practices	Operational performance	Organizational abandonment	Organizational change process
Employee retention					
HRM practices	0.697				
Operational performance	0.773	0.663			
Organizational abandonment	0.6	0.47	0.475		
Organizational change process	0.767	0.764	0.622	0.573	

Source: developed by authors using SPSS v27.

**Table 7:** Path coefficients.

	Coefficients Path (c)	Standard Deviation	*t*-Statistics	*p*-Values	Hypotheses Validation
HRM practices → Employee retention (H1)	0.355	0.052	6.851	0.000	H1 validated
HRM practices → Operational performance (H1)	0.449	0.047	9.573	0.000	
HRM practices → Organizational abandonment (H1)	−0.162	0.049	3.295	0.001	
HRM practices → Organizational change process (H2)	0.682	0.027	25.697	0.000	H2 validated
Organizational change process → Employee retention (H3)	0.467	0.048	9.748	0.000	H3 validated
Organizational change process → Operational performance (H3)	0.245	0.055	4.430	0.000	
Organizational change process → Organizational abandonment (H3)	−0.387	0.036	10.898	0.000	

Source: developed by authors using SPSS v27.

**Table 8:** Specific indirect and total effects.

		Coefficients Path (c)	Standard Deviation	*t*-Statistics	*p*-Values	Mediating Effect	Hypotheses Validation
Specific indirect effects	HRM practices → Organizational change process → Organizational abandonment (H4)	−0.264	0.027	9.934	0.000	Strong	H4 validated
	HRM practices → Organizational change process → Operational performance (H4)	0.167	0.038	4.355	0.000	Moderate	
	HRM practices → Organizational change process → Employee retention (H4)	0.318	0.034	9.312	0.000	Moderate	
Total effects	HRM practices → Employee retention (H4)	0.673	0.033	20.495	0.000		
	HRM practices → Operational performance (H4)	0.615	0.029	21.143	0.000		
	HRM practices → Organizational abandonment (H4)	−0.426	0.042	10.101	0.000		

Source: developed by authors using SPSS v27.

## Data Availability

Not applicable.
